# The Nature of Activatory and Tolerogenic Dendritic Cell-Derived Signal 2

**DOI:** 10.3389/fimmu.2013.00198

**Published:** 2013-07-16

**Authors:** Francesca Granucci, Manfred B. Lutz, Ivan Zanoni

**Affiliations:** ^1^Department of Biotechnology and Bioscience, University of Milano-Bicocca, Milan, Italy; ^2^Institute of Virology and Immunobiology University of Wuerzburg, Wuerzburg, Germany

One of the most interesting issues in immunology is how the innate and adaptive branches of the immune system cooperate in vertebrate organisms to respond and destroy invading microorganisms without destroying self-tissues. More than 20 years ago, Charles Janeway proposed the innate immune recognition theory (1). He hypothesized the existence of innate receptors (Pattern recognition receptors, PRRs) that, by recognizing molecular structures associated to pathogens (PAMPs) and being expressed by antigen presenting cells (APCs) and epithelial cells, could alert the immune system to the presence of a pathogen, making it possible to mount an immediate inflammatory response. Moreover, by transducing the alert signal in professional APCs and inducing the expression of costimulatory molecules, these receptors could control the activation of lymphocytes bearing clonal antigen-specific receptors, thereby promoting appropriate adaptive immune responses. Since adaptive immunity can be activated also following sterile inflammatory conditions, it was subsequently proposed by Polly Matzinger that the innate immune system could be also activated by endogenous danger signals, generically called danger associated molecular patterns (DAMPs) (2). The first prediction has been amply confirmed by the discovery of Toll-like receptors (3–5) and cytoplasmic PRRs such as RIG-like receptors (6). Other PRR families such as the NOD-like receptors and C-type lectins exert immunogenic or tolerogenic signals (7–9) and may recognize not strictly pathogens but also endogenous danger signals (10, 11).

Dendritic cells (DCs) have been identified as the cells of the innate immune system that, by sensing PAMPs or DAMPs transduce signals to the nucleus. This leads to a transcriptional reprograming of DCs with the consequent expression of three signals, namely signal 1 (MHC + peptide), signal 2 (surface costimulatory molecules), and signal 3 (cytokines) necessary for the priming of antigen-specific naïve T cell responses (signal 1 and 2) and T cell polarization (signal 3). The reason why DCs are superior with respect to other professional APCs in naïve T cell activation has not been unequivocally defined. It has not been established whether DCs can provide a special “signal 2” or simply very high levels, compared with other APCs, of commonly expressed signals 1 and 2, so that a naïve T cell could reach the threshold of activation.

A second aspect of DC biology needs also to be taken into account. Concerning the question of how self-tissues are not destroyed following the initiation of adaptive immune responses, different mechanisms of central and peripheral auto-reactive T cell tolerization have been proposed (12). In particular, it has been defined that high affinity T cells are deleted in the thymus, while low affinity auto-reactive T cells or T cells specific for tissue-sequestered antigens that do not have access to the thymus are controlled in the periphery. In a simplified vision of how peripheral T cell tolerance could be induced and maintained, it was thought that, in resting conditions, immature DCs, expressing low levels of signal 1 and low or no levels of signal 2, were able to induce T cell unresponsiveness. Nevertheless, it is now clear that a fundamental contribution to the peripheral tolerance is due to the conversion of naïve T cells into peripheral regulatory T cells (pTreg cells) and it is also clear that DCs need to receive a specific conditioning to become able to induce pTreg cell differentiation. Even more intriguing is that also DCs activated through PRRs, with particular Toll-like receptor (TLR) agonists, are capable of generating pTreg cell conversion if these agonists induce the production of the appropriate cytokines. Thus, what is emerging is that immature DCs are not able to induce tolerance by default but need to receive specific signals in order to acquire the ability of transferring to T cells a tolerogenic, rather than an activatory, signal 2.

Given these premises, this special issue covers the following topics:

1. The responses induced specifically in DCs by PAMPs and DAMPs and the consequences of these responses.
2. The DAMP and PAMP receptors expressed by different DC subsets and the consequences in the activation of adaptive immune responses.
3. How DCs induce and maintain peripheral T cell tolerance and the stimuli that confer tolerogenicity.
